# Editorial Notes and Miscellany

**Published:** 1912-01

**Authors:** 


### Editorial Notes and Miscellany.

---

DR. R. L. GRAHAM, Cotulla, Texas, offers for sale an established practice of $3500 and a residence worth $3250. Here is a fine opening for someone. Write to the doctor.

---

CLUB.—TEXAS MEDICAL JOURNAL, one year, $1.00; *American Journal Clinical Medicine,* $2.00; will send both journals for $2.00; will add Dr. Daniel's book, "Dr. Bruno," for $1.00 more. Here you get $3.00 for $2.00, or $4.50 for $3.00. Renewals count if arrears are paid.

---

DR. RALPH STEINER, State Health Officer and President State Board of Health, is distributing to the county health officers free about $20,000 worth of diphtheria antitoxine. He has taken the stump also in the crusade against ye hookworm and lectured at Waco December 27th ult.

---

WACO, TEXAS, December 14, 1911.

*Dr. F. E. Daniel, Austin, Texas.*

DEAR DOCTOR: I herewith hand you one dollar bill for the best journal I know, the "Red Back." I have been a reader of your journal so long that I would not think of doing without it.

W. L. CROSSTHWAITE, M. D.

---

THE NEW YORK MEDICAL JOURNAL is to be congratulated upon having secured the services as editor of Dr. Charles E. de M. Sajous. Dr. Sajous is, in the opinion of this journal, by far the ablest medical scholar in America, a man of extensive knowledge and broad cultivation in other branches of literature than medicine, a beautiful and polished writer and the author of several text-books and monographs. He is a worthy successor of the lamented

Dr. Frank P. Foster, who graced the tripod of the *New York Medical Journal* nearly forty years.

The value of Dr. Sajous' services to medical science has been recognized in France by his being made a member of the Legion of Honor, while in Belgium he received the order of Leopold and was made a Knight Commander of the Liberator, besides receiving other titles, both governmental and scientific. In America Dr. Sajous has been president and vice president of many societies and is a fellow of the College of Physicians of Philadelphia and of the American Philosophical Society. He brings to bear on the editorial problems of the *New York Medical Journal* a brilliant and well informed mind, wide experience, and a thorough knowledge of the needs of the American physician.

